# Long-Term Moderate-Level Noise Exposure Caused Hyperexcitability in the Central Auditory System

**DOI:** 10.1155/np/8842073

**Published:** 2025-01-24

**Authors:** Fei Xu, Guangdi Chen, Li Li, Wei Sun

**Affiliations:** ^1^Department of Communicative Disorders and Sciences, State University of New York at Buffalo, Buffalo, New York, USA; ^2^Department of Hearing and Speech Sciences, Zhejiang Chinese Medical University, Hangzhou, China

## Abstract

Noise exposure is one of the most common causes of hearing loss and hyperacusis. Studies have shown that noise exposure can induce a cortical gain to compensate for reduced input of the cochlea, which may contribute to the increased sound sensitivity. However, many people with hyperacusis have no measurable cochlear lesion after being exposed to loud sound. In this experiment, we studied the neurological alterations in the cortical and subcortical areas following a prolonged moderate level of noise exposure (84 dB SPL, 8 h/day for 4 weeks) in the laboratory mice. The cochlear function was monitored by auditory brainstem responses (ABRs). The behavioral auditory sensitivity and temporal processing were evaluated using the acoustic startle response (ASR) and gap-induced prepulse inhibition (gap-PPI). The central auditory functions were determined by electrophysiological recordings of the inferior colliculus (IC) and the auditory cortex (AC). Our results showed that although there was no significant difference in the ABR thresholds, the noise group showed enhanced ASR and gap-PPI compared to the control group. Increased neural activity in both the IC and the AC was recorded in the noise-exposed mice compared to the control group, suggesting a central gain in both the subcortical and cortical regions. The current source density (CSD) analysis of the AC response revealed an increased columnar excitation and reduced corticocortical projection in the noise group, different from the central gain model of noise-induced hearing loss. Our results suggest that chronic “nondestructive” noise can increase the gain of the central auditory system by altering the balance of auditory thalamocortical and intracortical inputs, which may contribute to the increased sound sensitivity in people with normal hearing.

## 1. Introduction

Environmental noise is a common cause of hearing impairments [1]. It is well acknowledged that exposure to a high-intensity sound can severely damage the cochlea and cause auditory disorders, including tinnitus and hyperacusis. Tinnitus, ringing in the ear, is a common disorder affecting people's lives after noise exposure. It has been estimated that the prevalence of tinnitus in the United States is about 27 million in 2014 [2]. Many people with tinnitus also have reduced sound tolerance, known as hyperacusis. One of the mechanisms of tinnitus and hyperacusis is that hearing loss can enhance the response in the central auditory system to compensate for reduced peripheral input to maintain its activity, referred to as the central gain increase [3, 4]. Lots of evidence on cortical plasticity have been revealed to support this mechanism. However, many tinnitus and hyperacusis subjects may have no measurable hearing loss [5]. Whether the central gain can be induced without severe damage on cochlea has not been studied.

The National Institute of Occupational Safety and Health (NIOSH) recommended workplace noise exposure standards of 85 dB SPL to avoid harmful consequences. Although noise below 85 dB SPL may not cause severe damages to the cochlea, numerous studies have shown that continued exposure to a noise lower than 85 dB SPL can still cause changes in auditory processing along the auditory pathway [6–8]. For example, exposure to broadband noise at 84 dB SPL can increase the activity of the dorsal cochlear nucleus (DCN) [9]. A recent study also found prolonged noise exposure to low-level noise caused anxiety behavior and hyperexcitability in the amygdala in mice [10]. The hyperexcitability in the central auditory system may be correlated to hyperacusis [11]. Therefore, whether the chronic exposure of low-intensity noise is “safe” or has a relevant impact on the auditory pathway is an open question. Currently, “safe” noise was typically referred to the acute damage in the cochlea. Whether there was a chronic impact on the central auditory system or a relevant psychophysical impact on the facilitation of sensory or cross-modal integration has rarely been considered.

There are many of these models, a moderate level of hearing loss was induced, and subcortical activity was lower compared to the control group [12]. While these data primarily suggest extensive involvement of many nuclei along the auditory pathway and off-cortical projections reflecting and compensating for impairments in the auditory periphery, relatively little is known about the convergent contributions of thalamocortical and intracortical inputs to these effects. In previous studies, we developed a method to dissociate the thalamocortical from intracortical contributions to cortical activity. The method is based on the analysis of the residue of the current source density (CSD) reconstructed from measurements of the local field potential along linear electrode arrays penetrating the cortical layers perpendicular to the cortical surface. The method is highly sensitive and allows evaluation of the relative contributions of thalamocortically and intracortical relayed activity to a given level of stimulation in the cortex. Here, we used this approach to investigate the auditory temporal processing and cortical–subcortical function following chronic noise exposure at a moderate level.

## 2. Material and Methods

### 2.1. Animals and Noise Exposure

**Figure 1:**
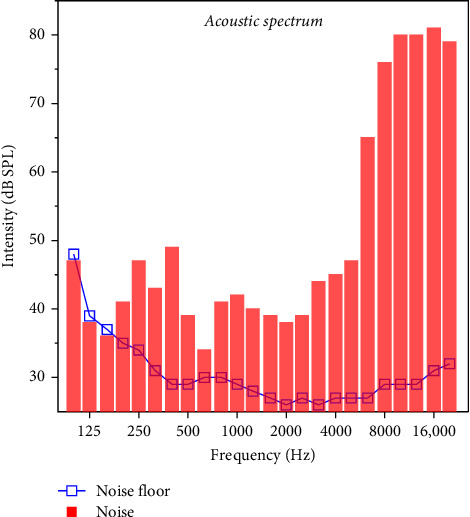
The spectra of the noise and room background noise. The energy of noise is above 6 kHz (note: the sound level meter can only measure below 24 kHz due to the sampling rate), and the background noise is at low frequencies (<250 Hz).

CBA mice, a mouse stain that can maintain normal hearing threshold until 24 months, were used in this study. Mice at the age of 8 weeks (both males and females) were obtained from Jackson Labs and randomly assigned into the noise group (*n* = 10) or the control group (*n* = 10). The mice of the noise exposure group were exposed to a moderate level of broadband noise (MLN) at 84 dB SPL for 8 h a day (10 pm to 6 am) for 4 weeks. The broadband noise (6–60 kHz) was generated by Adobe Audition. Sound was produced by the computer sound card amplified by a NX1000 2-channel power amplifier (Behringer) and delivered through an FT17H (Fostex) speaker placed 10 cm above the mice. To ensure a steady noise circumstance, acoustic levels were evaluated before and after each exposure using a sound level meter (Larson Davis). The spectra of the noise and room background noise were shown in Figure 1. The energy of noise is at above 6 kHz (note: the sound level meter can only measure below 24 kHz due to the sampling rate). The control group animals were raised in an environment with a background noise level of no more than 60 dB SPL, and major energy is at low frequencies (<250 Hz). All experimental procedures were reviewed and approved by the Institutional Animal Care and Use Committee of State University of New York at Buffalo.

### 2.2. Hearing Evaluation

The hearing thresholds of the mice were evaluated using the auditory brainstem response (ABR). Mice were anesthetized with a mixture of ketamine (80 mg/kg, i.p.) and xylazine (6 mg/kg, i.p.), and their body temperatures were maintained at 37°C using a thermally regulated heating pad system (Harvard Apparatus, Cambridge, MA). Alternating phase tone bursts (5-ms duration, 1-ms rise/fall time, Hanning window) of 4, 8, 16, and 32 kHz were generated at a sampling rate of 200 kHz. The tone bursts were presented through an MF1 loudspeaker 10 cm away from the right ear. Stimuli were presented at a rate of 21.6/s, and the sound levels varied from 10 to 90 dB SPL (5–10 dB steps).

### 2.3. Acoustic Startle Response (ASR) and Gap-Induced Prepulse Inhibition (Gap-PPI) Test

ASR and gap-PPI were recorded following the procedure described in our previous paper [13]. Mice were placed in a small wire mesh cage (4 cm × 3.5 cm × 7 cm) to restrict their movement within a calibrated sound field. The cage was put into a chamber and mounted on a plexiglass base that rested on a sensitive piezoelectric transducer. The interior of the chamber was insulated with a 1-inch-thick layer of dense foam material to reduce sound reflections. The output of the piezo transducer was amplified and filtered using a low-pass filter (LPF-300, World Precision Instruments, Sarasota, FL, USA) and then connected to an A/D converter on an RP2 real-time processor (TDT). The ASR-eliciting stimuli consisted of narrowband noise bursts (50-ms duration, 1 ms rise/fall time, 2 kHz bandwidth) centered at 8 kHz, presented at 100 dB SPL. The interstimulus interval (ISI) randomly varied from 18 to 22 s.

To detect gap-PPI, the startle responses were recorded with/without silent gaps embedded in a background broadband noise (1–20 kHz) at 60 dB SPL. The duration of the silent gap varied from 1 to 16 ms (1, 2, 4, 6, 8, and 16 ms), and the gap was presented 160 ms before the startle stimulus. A recording window of 180 ms after the onset of the startle sound was used to measure the startle response. The following formula was used to calculate the gap-PPI ratio: $\text{gap-PPI ratio}=\left(\frac{{\text{ASR}}_{\text{gap}}}{{\text{ASR}}_{\text{no gap}}}\right)$. The low ratio means high inhibition of the startle reflex, which suggests better gap detection. All the different sound conditions were intermixed, and their order was double randomized within and across trials for each test session.

### 2.4. Electrophysiological Recordings

The local field potentials and unit activities were recorded from the anesthetized mice from the right side of the inferior colliculus (IC) and the auditory cortex (AC), contralateral side to the sound stimuli. A mixture of ketamine (100 mg/kg, i.p.) and xylazine (10 mg/kg, i.p.) was used for anesthesia. The pedal withdrawal reflex of the hind limbs was checked every 25 min to assess the anesthetic depth. Mouse body temperature was maintained at 37°C using a thermally regulated heating pad system (Harvard Apparatus, Cambridge, MA). A small dose of supplemental ketamine and xylazine mixture (~0.1 ml) was added every 30 min during the recording. A custom fixer was used to firmly hold the mouse's palate and head during the test. A 16-channel microelectrode (NeuroNexus, A1 × 16−10 mm-100e177, Ann Arbor, MI) was mounted on a hydraulic manipulator (FHC Inc., Bowdoinham, ME). A stainless steel electrode inserted into the neck was used as the ground. The output of the electrode was connected to a 16-channel preamplifier (RA16PA, Tucker-Davis Technology, TDT). The output of the preamplifier was delivered to a digital signal processing module (RZ5, TDT) connected to a computer. The multiunit spike discharges were recorded using OpenEx (TDT) and custom software.

For the IC recordings, a 3 × 3-mm region of cranial bone overlying the dorsocaudal aspect of the right side of cerebellum was removed to expose the IC [14]. Noise bursts (50 ms, 80–90 dB SPL) were presented three times per second as the electrode was advanced into the IC to search for neurons. Recordings were obtained from different regions of the central nucleus of the IC; this was accomplished by inserting the electrode into different locations starting from the lateral side and moving medially while avoiding major blood vessels. As the best frequency (BF) of IC neurons increased with depth from the IC surface, the electrode was advanced to 1–1.2 mm in all the penetrations, and multiunit firing response was recorded. The depth of recording sites was normalized based on the most superficial recording sites that achieved good signals. The BF from each recording site was defined as the frequency with the greatest response level.

For the AC recordings, insertion was started at 2 mm posterior to bregma and 2.5 mm lateral to midline. Three to four penetrations were repeatedly performed by moving the electrode caudally at a 0.2–0.3 mm step while avoiding major blood vessels. The electrode advanced at a speed of about 5 μm per second until 1.2 mm in the AC. A broadband noise burst (80–90 dB SPL) was used as a searching sound to monitor neural responses, and recording was performed for 20–25 min. The electrode was moved laterally and caudally in a 0.2–0.3 mm step to cover the whole AC. The primary AC was confirmed based on sharp initial negative LFP peak, multiunit activity (MUA), specifically short latency responses, and excitatory frequency response area (eFRA) [14, 15].

The IC and AC responses were elicited by 50 ms tone bursts to the contralateral ear (1 ms rise/fall time, 4–64 kHz, and 20 logarithmically spaced steps) to generate peristimulus time histograms (PSTHs) of MUA and field potential. The sound intensities were varied from 10 to 90 dB SPL in 10 dB steps. Sound stimuli were generated with the TDT System-3 hardware and presented through a multifield magnetic speaker (MF1, TDT). The sound intensity was calibrated using a sound level meter (824, Larson Davis, Depew, NY) with a 1/4-inch condenser microphone (Larson Davis). Spontaneous activity was calculated by tallying the number of spikes within the last 500 ms of each trial and then calculating the average spontaneous firing rate across all trials.

### 2.5. Residual CSD Profile Analysis

The CSD is essentially a global activity caused primarily by excitatory synapses; the vertical depth of each sink and its corresponding source (or sources) indicates the area where the activated cell extends its dendrites [16]. The one-dimensional CSD profiles were calculated from the second spatial derivative of the LFP [16–18]. Current sinks have been interpreted as depolarizing events and can reveal patterns of current influx, whereas current sources generally reflect passive return currents. Based on laminar local field potentials recorded from the AC, CSD analysis was performed with custom-written scripts in MATLAB (R2020b; The MathWorks). To visualize the CSD profiles, we utilized color image plots, which were generated through linear interpolation along the depth axis, with red and blue representing current sinks and sources, respectively. Green represents an approximate value of zero:

(1) $$\begin{array}{c} -\text{CSD}\approx \frac{\delta^{2}\Phi \left(z\right)}{\delta z^{2}}=\frac{\Phi \left(z+n\Delta z\right)-2\Phi \left(z\right)+\Phi \left(z-n\Delta z\right)}{{\left(n\Delta z\right)}^{2}}, \end{array}$$

where *Φ* is the field potential, *z* is the spatial coordinate perpendicular to the cortical layer, *Δ*z is the sampling interval (100 μm), and *n* is the differentiation grid. Equation (1) only considers one dimension and assumes isotropic conductance.

The averaged rectified CSD (AVREC) [19, 20] was calculated by averaging the absolute values of the CSD separately across the number of channels for each recording trial. Subsequently, data were averaged across trials (Equation 2). The relative residues (RelRes) of CSD [21] were calculated as the sum of the CSD values over the number of recording channels divided by the sum of the respective absolute values (Equation 3):

(2) $$\begin{array}{c} \text{AVREC}=\frac{\sum_{i=1}^{n}\left|{\text{CSD}}_{i}\right|\left(t\right)}{n}, \end{array}$$

(3) $$\begin{array}{c} \text{Relative residues}=\frac{\sum_{i=1}^{n}{\text{CSD}}_{i}\left(t\right)}{\sum_{i=1}^{n}\left|{\text{CSD}}_{I}\right|\left(t\right)}. \end{array}$$

### 2.6. Statistical Analysis

Graphs were generated with GraphPad (Prism 8.3.0 for Windows, GraphPad Software, San Diego, CA). All data were expressed as mean ± standard error of the mean (SEM). Statistical tests performed by the GraphPad Software. Unpaired Student's *t*-tests were used for the data analysis. Differences were considered statistically significant when *p*-values were smaller than *α* = 0.05.

## 3. Results

### 3.1. ABR Threshold in the Noise-Exposed Mice

**Figure 2:**
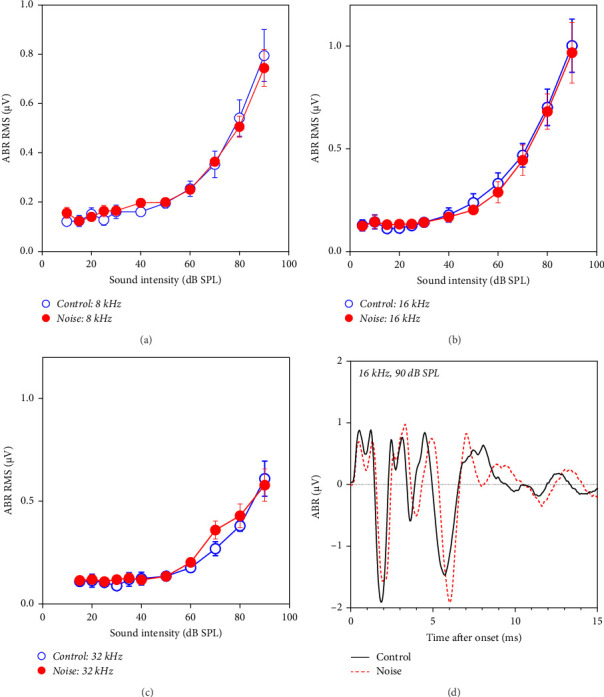
Amplitude–intensity functions of auditory brainstem response (ABR). The amplitude was calculated in the root mean square (RMS) of ABR in a 0–8-ms window. The amplitude of the noise group (*n* = 4) and the control group (*n* = 4) has no difference at 8, 16, and 32 kHz (A–C). Typical ABR waveform elicited by 16 kHz tone burst presented at 90 dB SPL from the control (black solid line) and noise group (red dashed line) was shown in (D).

To determine if long-term noise exposure at 84 dB SPL caused any damage to hearing threshold, ABR testing was performed in the control group and the noise group. There were no differences in ABR thresholds between the control group and the noise group (right after removing from the noise) at 4, 8, 16, and 32 kHz (Figure 2A–C). The typical ABR waveforms of mice in the control and noise groups were shown in Figure 2D (80 dB SPL). Differentiated waveforms (wave I–V) were shown in both groups, and there was no significant difference found in both groups.

### 3.2. Noise-Exposed Mice Showed Increased ASR and Gap-PPI

**Figure 3:**
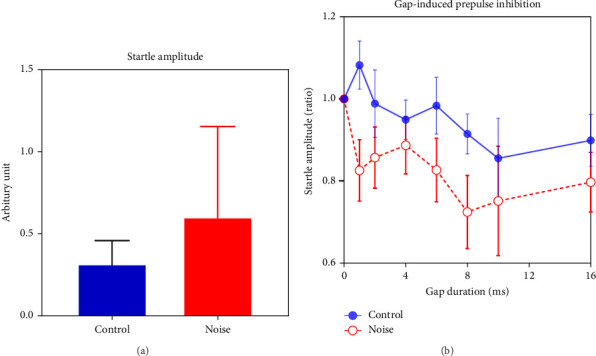
Auditory sensitivity was evaluated by acoustic startle response (ASR) and gap-induced prepulse inhibition (gap-PPI). (A) The noise group showed a larger ASR response than the control group. (B) The noise group (*n* = 4) showed enhanced gap (1, 2, 4, 6, 8, 10, 16 ms) induced inhibition than the control group (*n* = 4).

The mean amplitude of ASR in the noise group was higher than in the control group (Figure 3A). However, the difference was not statistically significant due to the large intragroup variations. Gap-PPI was measured and calculated to identify changes in temporal acuity following moderate-level noise exposure. A significant reduction in gap-PPI was found in the noise group compared to the control group. The differences in the startle ratio (gap/no gap) at 6, 8, and 10-ms gap durations are significant between the two groups (Figure 3B, two-way ANOVA, *p* < 0.05), indicating improvement for short gap detection following noise exposure.

### 3.3. Noise Exposure Caused Enhanced Responses in the IC

**Figure 4:**
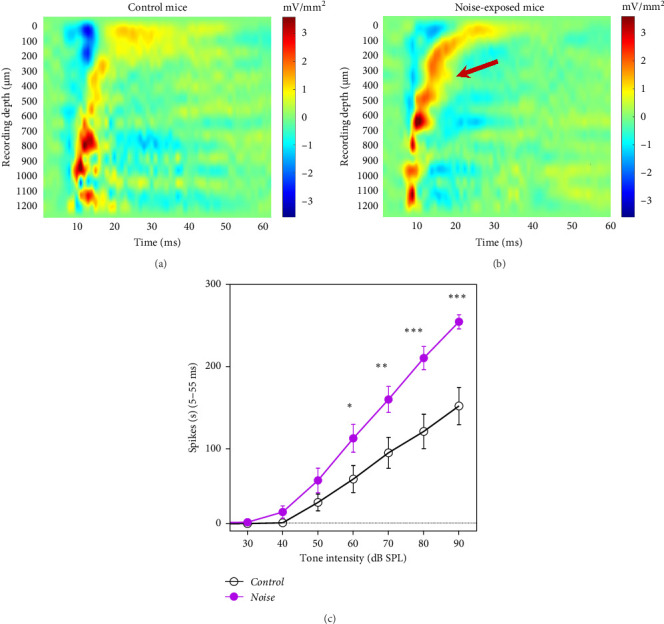
Long-term moderate noise exposure changes inferior colliculus (IC) response patterns and enhances best frequency (BF) activities. Color maps of representative laminar current source density (CSD) profile of recording sites of the IC site of control (A) and noise-exposed mice (B). The prominent current sinks (red) are reflective of a depolarization of neurons in the surrounding region of the 16-channel linear microelectrode array, whereas prominent current sources (blue) reflect repolarization of neurons in the surrounding regions. The noise group shows an expanded field and increased amplitude of sound-evoked sink at the middle and upper layers (arrow). (C) The mean (± standard error of the mean [SEM]) firing rates of the noise group (purple-filled circles, *n* = 24) was significantly higher than the control group (black open circles, *n* = 30) from 30 to 90 dB SPL. The firing rates were calculated at frequencies ±1 octave centered at the BF of the recorded neurons (*⁣*^*∗*^*p* < 0.05, *⁣*^*∗∗*^*p* < 0.01, and *⁣*^*∗∗∗*^*p* < 0.001, paired Student's *t*-test).

CSD analysis was used to investigate how long-term moderate-level noise exposure affects laminar tone-evoked processing in the IC. A typical CSD profile of a control and a noise-exposed mouse was shown in Figure 4A,B, respectively. As previously reported, the central region of the IC has a continuous sink distribution with a decreased responding latency pattern to the recording [22]. The prominent current sinks (red) are reflective of the depolarization of neurons in the surrounding region of the 16-channel linear microelectrode array, whereas prominent current sources (blue) reflect a repolarization of neurons. The noise group shows an expanded field and increased amplitude of sound-evoked sink at the middle and upper layers (Figure 4B, arrow). The neurons in the upper and middle layers have lower best frequencies and longer latencies compared to the deeper layers.

The mean spike rate of the IC response was calculated in a 5–55 ms window after the onset of sound stimuli. The input–output function of the IC responses centered at their BFs (±1 octave bandwidth) in the control group (32 MUA recorded from 4 mice) and noise group (24 MUA recorded from 4 mice) was calculated (Figure 4C). Averaged firing rates recorded from ICs of the noise group were significantly higher than the control group at 60–90 dB SPL (*p* < 0.01, Student's *t*-test). These findings indicate a hyper-responsiveness to acoustic stimulation of neurons in the IC of mice exposed to a chronic noise at 84 dB SPL.

### 3.4. MUA in the AC

**Figure 5:**
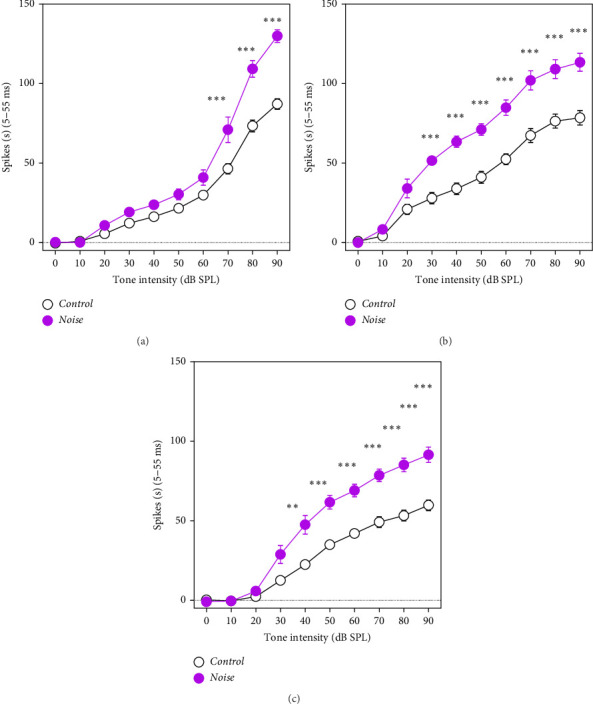
Mean (± standard error of the mean [SEM]) multiunit spike discharge rates were recorded and computed in 5–55-ms time windows from the auditory cortex (AC) in control (black line, 564 neurons recorded 6 mice) and noise-exposed mice (purple line, 592 recordings from 50 penetrations of 6 mice) at (A) 4–8 kHz, (B) 12–16 kHz, and (C) 20–35 kHz as a function of stimulus intensity, which revealed the effect of noise exposure on AC I/O function. Compared to control mice, the firing rate of AC in noise-exposed mice was significantly enhanced at high intensities of 4 and 8 kHz, 12 and 16 kHz, and 20 and 35 kHz (*⁣*^*∗*^*p* < 0.05, *⁣*^*∗∗*^*p* < 0.01, and *⁣*^*∗∗∗*^*p* < 0.001).

The mean spike rate (5–55 ms after the onset of sound stimuli) of the AC response was used to compute the AC population responses at low, middle, and high frequencies (Figure 5A–C, 4–8, 12−16, and 20–35 kHz). Compared with the control group (564 recordings from 57 penetrations of 6 mice), the firing rate in AC of the noise group was significantly increased at high intensities (592 recordings from 50 penetrations of 6 mice).

**Figure 6:**
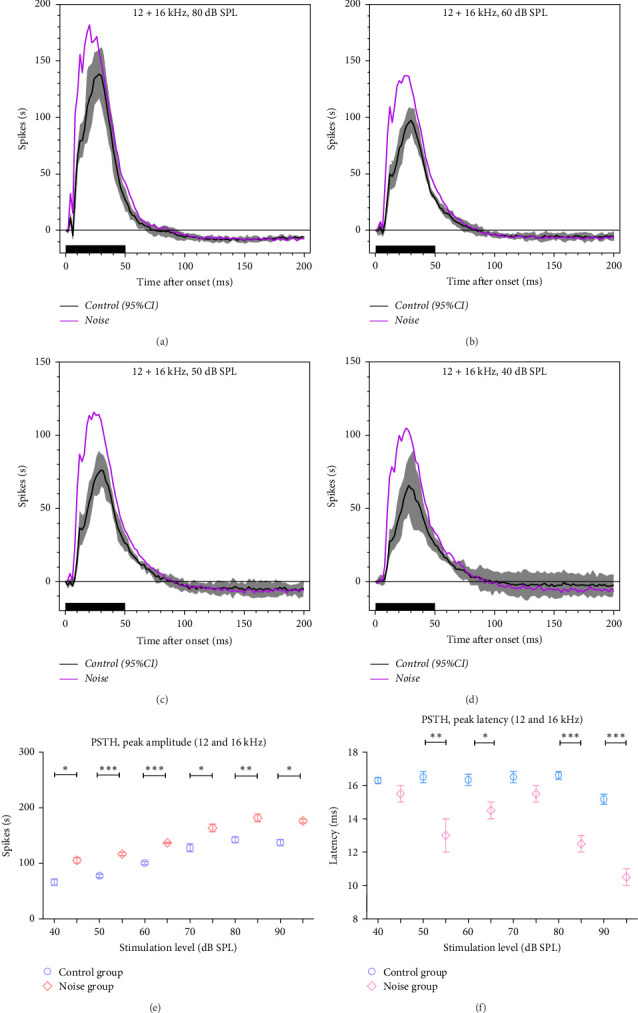
Mean population multiunit peristimulus time histograms (PSTHs, 200 ms window, 1-ms bin width, 50 ms tone bursts) obtained from the auditory cortex (AC) at 80- (A), 60- (B), 50- (C), and 40-dB SPL (D) (12 and 16 kHz) for the control (black line, 564 neurons recording from 6 mice) and noise exposure mice (purple line, 592 neurons recording from 6 mice). Shaded regions represent a 95% confidence interval (CI) obtained from the data of controls. The PSTH showed a peak at 20–30 ms. The average peak activity of noise-exposed mice raised outside the 95% CI means of the control mice. Noise-exposed mice exhibited higher average PSTH peak amplitudes (E) and shorter peak latencies compared with control mice (F). The poststimulus response (>100 ms) in the control mice fell below the spontaneous rate, referred to as the poststimulus inhibition. There is no difference in poststimulus inhibition between the two groups.

The mean PSTHs of the neural firing rate at different sound intensities in the two groups responding to the 50-ms tone burst were shown in Figure 6A–D (90–40 dB SPL, 10 dB step). The PSTHs were displayed in a 200-ms time window with 1-ms bin widths. The typical responses showed a sharp increase to the onset sound stimuli followed by a rapid decrease. The peak amplitude was higher in the noise group (176.3 ± 4 spikes/s, *n* = 6) compared to the control group (137.5 ± 5.9 spikes/s, *n* = 6). The differences were statistically significant from 90 to 40 dB (Figure 6E, *p*  < 0.05, paired Student's *t*-test). A shorter latency was found in the noise group at different intensities. The peak latency of the PSTHs increased from 15.17 ± 0.31 ms to 16.29 ± 0.18 ms in the control group, and the noise group increased from 10.5 ± 0.5 ms to 15.5 ± 0.5 ms with sound intensity decreased from 90 to 40 dB SPL. The differences were statistically significant at 90, 80, 60, and 50 dB (Figure 6F, *p*  < 0.05, paired Student's *t*-test). These data suggest that there was enhanced excitation of the AC in the noise group.

### 3.5. CSD Analysis of the AC Response

**Figure 7:**
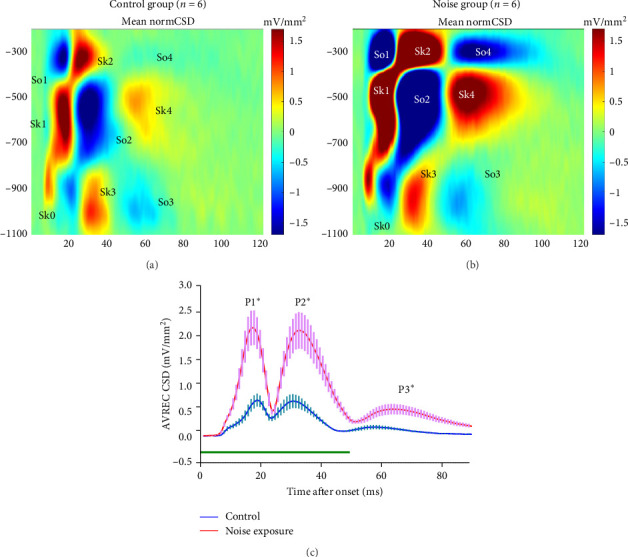
(A, B) The average current source density (CSD) profile of the control group (*n* = 6) and the noise group (*n* = 6). Five primary current sinks (Sk0–Sk4) and four sources (So1–So4) in the laminar CSD profiles are typically seen. Tone-evoked CSD profiles display afferent early sink activity (Sk1) in granular layer III/IV and infragranular layer Vb (Sk0), and subsequent activation of supragranular layer I/II (Sk2) and infragranular layers VI (Sk3), and the poststimuli activation of the granular layer (Sk4). Roman numbers indicate cortical layers. After long-term noise exposure, tone-evoked CSD profiles showed increased strength of the early afferent input in layer III and layer V and succedent stronger activation of supragranular layers I/II. (C) The average rectified CSD (AVREC) from the normalized CSD profile which was evoked by 40–90 dB. The noise group (red line, *n* = 6) showed higher peak1, peak2, and peak3 compared to the control group (blue line, *n* = 6); the differences were statistically significant (*⁣*^*∗*^*p*  < 0.05, paired Student's *t*-test).

The average CSD profiles of the noise group (*n* = 6) and the control group (*n* = 6) were shown in Figure 7. Both group mice exhibited a similar canonical CSD pattern, including four primary sinks (Sk1–Sk4) and four sources (So1–So4). In previous studies, the initial sinks Sk0 and Sk1 characterized by the shortest latencies were in the thalamic input layers V/VI and III/IV, respectively. These primary sinks are prominent and reliably evoked components in the CSD patterns observed across the analyzed stimuli. The current sinks in the laminar CSD profiles are typically seen as signifying excitatory occurrences like axonal depolarizations and synaptic activations, while current sources often represent passive return currents. The noise group showed enhanced excitation compared to the control group primarily at layer III/IV. The average AVREC amplitudes were shown in Figure 7C which shows three peaks (P1, P2, and P3) which represent early (Sk1–So1), middle (Sk2–So2), and late (Sk3–So3) sinks and sources. The noise group showed a significantly larger response in all short and long latency peaks (peaks 1–3), suggesting enhanced thalamocortical input and overall intracortical excitation in the AC.

**Figure 8:**
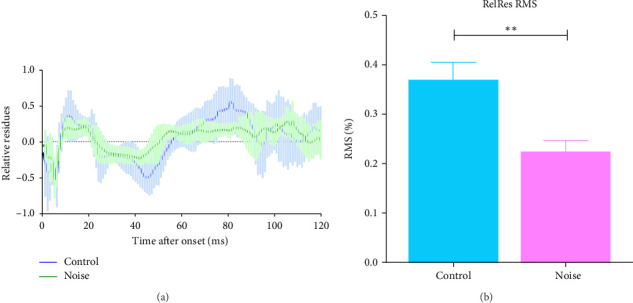
The relative residues (RelRes) of the current source density (CSD) of the auditory cortex (AC) response analysis showed a reduced response in the noise group (green curve, *n* = 6) compared to the control group (blue curve, *n* = 6). (A) The mean RelRel response profile (0–120 ms). (B) The RMS (± standard error of the mean [SEM]) of the RelRel response showed a reduced overall value (*⁣*^*∗∗*^*p* < 0.01, paired Student's *t*-test).

To quantify the unbalanced cortical contributions of sinks and sources of the CSD profile, the RelRes of CSD were analyzed by quantifying the root mean square (RMS) of the RelRes response. The RelRes analysis results were shown in Figure 8. Compared to the control group (*n* = 6), the RMS amplitude of RelRes response of the noise group (*n* = 6) was significantly lower, suggesting a less fluctuation in the RelRes response (*p* < 0.01, Student's *t*-test). As the RelRes is an indicator for the corticocortical spread of activity across columns, the result suggests a relatively lower cross-columnar activity in the noise group.

## 4. Discussion

In this study, we have revealed that long-term exposure to a moderate level of noise (84 dB SPL for 4 weeks) affects sound processing and neuronal activities in the IC and the AC. The three major findings of the study are as follows: (1) Consistent with our previous studies, the startle and gap-PPI were enhanced without permanent hearing threshold shift; (2) activities in the subcortical and cortical areas are increased by noise exposure; and (3) the CSD analysis of the AC response showed an increased intracolumnar response and decreased intercortical activity. Our results demonstrated that a “safe noise” for the cochlea can have an impact on auditory response and central auditory functions.

### 4.1. Noise Exposure on Auditory Processing

Many studies have shown that exposure to loud noise can cause enhanced ASR, even with lesions in the cochlea [23, 24]. These studies also showed impaired gap-PPI, indicating signs of tinnitus. These findings provide evidence that noise exposure may be a cause of tinnitus and hyperacusis. Our study further demonstrated that exposure to a moderate level of noise (84 dB SPL) can still cause increased sound sensitivity [8]. Surprisingly, our data found that gap detection was improved after 4 weeks of noise exposure (84 dB SPL). The cause of the improved gap-PPI is still unclear. Our previous studies found that noise exposure increased the number of c-Fos-labeled neurons in the DCN and caudal pontine reticular nucleus (PnC) but not at a higher level in the central auditory nuclei, suggesting that changed acoustical temporal processing presumably induced by increased excitability of auditory brainstem neurons. We think the results may be related to increased synaptic release in the central auditory pathway, such as the bush cells in the cochlear nucleus [9].

### 4.2. Hyperexcitability in the IC and the AC

Noise-induced central gain changes have been linked to tinnitus and hyperacusis [3, 12]. One of the explanations is that the central auditory system compensates for peripheral damage. In most of those studies, the response in the IC was reduced [12]. In our study, we found hyperexcitability in the IC and the AC in the current model without cochlear damage. Our data suggest that prolonged chronic MLN exposure causes acute changes in subcortical inputs and corticocortical circuits in the AC. The results suggest that long-lasting adaptive processes adjust cortical processing over weeks to the altered sensory inputs, and the central gain does not rely on the peripheral damage.

Long-term noise exposure is likely leading to the formation of neural plasticity which may be helpful to alleviate auditory disorder after peripheral damage. For example, sound therapy is a common procedure for tinnitus and hyperacusis treatment [25–27]. Low-level noise can also enhance weak signal detection, a phenomenon known as stochastic resonance. Previous studies found that random noise (50–90 dB SPL) provided a cross-modal signal processing benefit [28].

### 4.3. Corticocortical Activity Decreases After Noise Exposure

**Figure 9:**
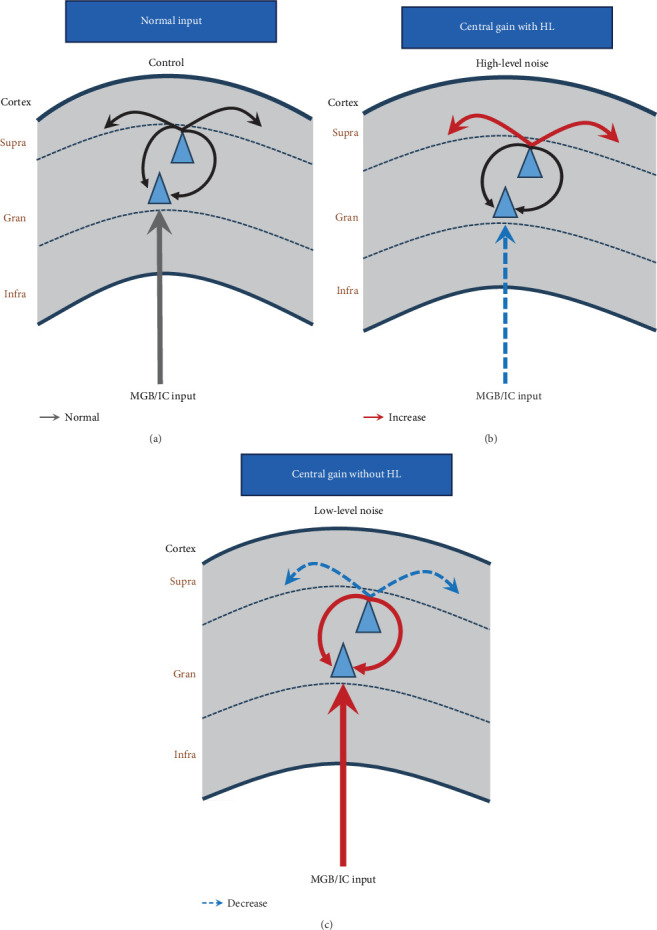
The simulated schematic diagram demonstrates the intracortical functional changes in the auditory cortex (AC) response caused by loud and moderate noise exposure. The black arrows represent normal projection, the red arrows represent increased projections, and the blue dash arrows represent decreased projections. (A) The gray dashed lines represent the boundary of the supragranular layer, granular layer, and infragranular layer boundaries. The uppermost arrows represent lateral corticocortical connections, the lowest up arrow represents the thalamocortical inputs, and the circle arrow represents local intracolumnar connections in the granular layer. (B) Noise trauma induced decreased strength of local thalamic input to the AC and increased corticocortical activity. (C) Moderate-level noise exposure showed increased thalamocortical inputs and intracolumnar projections and decreased the cross columnar projections.

A better understanding of the circuit mechanisms underlying this dynamic temporal process may provide new implications to ameliorate the effects of hyperacusis and tinnitus. The relative contributions of thalamocortical and intracortical pathways to cortical plasticity following noise exposure remain unclear. Previous studies have found that noise trauma caused enhanced corticocortical horizontal processes to compensate for the reduced overall cortical activation [29]. After 115 dB SPL 2 kHz sine wave sound exposure for 75 min, they found that despite a reduction in overall cortical activation after acoustic trauma, the RelRes CSD was elevated, suggesting a decreased thalamic input and an increased central gain mainly caused by intracortical projection. Our CSD and RelRes analysis provides information on intercolumnar corticocortical inputs and intracortical transmitted in layer III/IV. Our main finding is that after long-term moderate noise exposure, although the overall AC activation increased significantly, the RelRes CSD was significantly reduced, suggesting the contribution of corticocortical horizontal input was decreased. Our results suggest that an overcompensating mechanism for altered inputs in the AC is dependent on balanced alterations in thalamocortical and intracortical inputs, and the central gain was not found to be contingent upon peripheral damage. Through the studies above, we proposed a new cortical model of central gain (Figure 9A). In the central gain model caused by high level of noise exposure, the input from the thalamus or IC was decreased due to sound trauma, but the relative contribution of corticocortical activity was increased immediately after the trauma [29] (Figure 9B). In our moderate-level noise model, increased input from the thalamus was coupled with an increased local intracolumnar gain but decreased corticocortical activity. The increased gain was limited to local intracolumnar circuits and was not broadcast to wide-spread corticocortical circuits (Figure 9C). It seems likely that increased thalamic activation of ACx is the result of mechanisms of compensatory, homeostatic plasticity.

## 5. Conclusions

Our study found chronic exposure to a moderate level of noise caused hyperexcitability in both the IC and the AC, suggesting the central gain increase was not dependent on the cochlear damage. We also found the central gain increase in both the subcortical and cortical levels which may contribute to increased sound sensitivity.

## Data Availability

The data that support the findings of this study are available from the corresponding author upon reasonable request.
